# Oral vitamin C restores endothelial function during acute inflammation in young and older adults

**DOI:** 10.14814/phy2.15104

**Published:** 2021-11-11

**Authors:** Elizabeth C. Lefferts, Brooks A. Hibner, Wesley K. Lefferts, Natalia S. Lima, Tracy Baynard, Jacob M. Haus, Abbi D. Lane‐Cordova, Shane A. Phillips, Bo Fernhall

**Affiliations:** ^1^ Department of Kinesiology and Nutrition University of Illinois at Chicago Chicago Illinois USA; ^2^ Department of Kinesiology Iowa State University Ames Iowa USA; ^3^ School of Kinesiology University of Michigan Ann Arbor Michigan USA; ^4^ Department of Exercise Science Arnold School of Public Health University of South Carolina Columbia South Carolina USA; ^5^ Department of Physical Therapy University of Illinois at Chicago Chicago Illinois USA

**Keywords:** antioxidant, ascorbate, flow‐mediated dilation, pulse wave velocity

## Abstract

Oxidative stress has been linked to reductions in vascular function during acute inflammation in young adults; however, the effect of acute inflammation on vascular function with aging is inconclusive. The aim of this study was to determine if oral antioxidant administration eliminates vascular dysfunction during acute inflammation in young and older adults. Brachial flow‐mediated dilation (FMD) and carotid‐femoral pulse wave velocity (PWV) were measured in nine young (3 male, 24 ± 4 yrs, 26.2 ± 4.9 kg/m^2^) and 16 older (13 male, 64 ± 5 yrs, 25.8 ± 3.2 kg/m^2^) adults before and 2‐h after oral consumption of 2 g of vitamin C. The vitamin C protocol was completed at rest and 24 h after acute inflammation was induced via the typhoid vaccine. Venous blood samples were taken to measure markers of inflammation and vitamin C. Both interleukin‐6 (Δ+0.7 ± 1.8 pg/ml) and C‐reactive protein (Δ+1.9 ± 3.1 mg/L) were increased at 24 h following the vaccine (*p* < 0.01). There was no change in FMD or PWV following vitamin C administration at rest (*p* > 0.05). FMD was lower in all groups during acute inflammation (Δ‐1.4 ± 1.9%, *p* < 0.01), with no changes in PWV (Δ‐0.0 ± 0.9 m/s, *p* > 0.05). Vitamin C restored FMD back to initial values in young and older adults during acute inflammation (Δ+1.0 ± 1.8%, *p* < 0.01) with no change in inflammatory markers or PWV (*p* > 0.05). In conclusion, oral vitamin C restored endothelial function during acute inflammation in young and older adults, with no effect on aortic stiffness. The effect of vitamin C on endothelial function did not appear to be due to reductions in inflammatory markers. The exact mechanisms should be further investigated.

## Graphical abstract

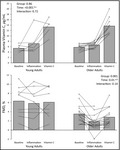

## 1 INTRODUCTION

Acute inflammation in young adults consistently elicits transient reductions in endothelial function (Hingorani et al., 2000a; Lane‐Cordova, Phillips, et al., 2016; Wallace et al., 2010) and may increase arterial stiffness (Vlachopoulos et al., 2005; Wallace et al., 2010), although this finding is not universal (Lane et al., 2015; Schroeder, Lefferts, et al., 2019). The inflammatory process can induce oxidative stress from the generation of excess reactive oxygen species, which lowers the antioxidant capacity of a system (El Assar et al., 2013). Increased oxidative stress may therefore explain the vascular responses seen during acute inflammation as it can reduce bioavailability of the potent vasodilator nitric oxide and subsequently induce endothelial dysfunction and functionally stiffen large, elastic arteries (Jain et al., 2014). Administration of a potent antioxidant (e.g., vitamin C infusions) has been shown to either partially (Clapp et al., 2004) or fully restore (Aschauer et al., 2014; Mittermayer et al., 2005; Pleiner et al., 2002, 2003) endothelial function during acute inflammation in young adults, suggesting reductions in endothelial function during acute inflammation are driven by oxidative stress. Whether antioxidant administration can also restore endothelial function in older adults is unknown but of great interest considering acute inflammation can increase cardiovascular event risk at least threefold in older adults (Corrales‐Medina et al., 2009, 2010, 2015; Smeeth et al., 2004).

Aging increases basal levels of oxidative stress (Donato et al., 2007, 2008), low‐grade systemic inflammation, and may alter vascular responses to acute inflammation. In contrast to young adults, older adults show less consistent vascular responses to acute inflammation. Some studies observe reductions in endothelial function and increases in arterial stiffness (Antoniades et al., 2011; Jae et al., 2013; Lane‐Cordova, Ranadive, et al., 2016), whereas others identify no such effect (Lane‐Cordova, Phillips, et al., 2016; Ranadive et al., 2014). Acute vitamin C infusion (Eskurza et al., 2004a; Moreau et al., 2005) and oral administration (Levine, Frie, et al., 1996; Raitakari et al., 2000) have been shown to improve endothelial function and arterial stiffness under resting conditions in older adults (i.e., acutely attenuate basal oxidative stress seen with aging). Oral vitamin C could therefore also improve endothelial function or arterial stiffness during acute inflammation in older adults and potentially serve as an easily accessible, prophylactic during acute inflammatory insults. The aim of this study was to determine if oral antioxidant administration eliminates vascular dysfunction during acute inflammation in young and older adults, assessed via flow‐mediated dilation and carotid‐femoral pulse wave velocity. We hypothesized antioxidant administration would restore vascular function to baseline values in young and older adults during acute inflammation.

## 2 METHODS

### 2.1 Study participants

Nine young (18–35 years) and 16 older (55–75 years) generally healthy adults were recruited from the local university and surrounding areas for participation. Participants were excluded for known cardiovascular, inflammatory, or metabolic disease; current use of >2 blood pressure medications or change in medication within the last 2 months; bleeding disorders; body mass index >30 kg/m^2^; current smoking; antioxidant or vitamin supplementation or use of anti‐inflammatory medication within the last 2 weeks; pregnancy; hormone therapy; illness or vaccination within the last 2 weeks; or a typhoid vaccination within the last 2 years. Young female participants were studied during the first 7 days of their menstrual cycle or during the placebo phase for those on oral contraceptives. All older females were post‐menopausal with no menstrual cycle reported in the last 1 year or more. This study was approved by the Institutional Review Board of the University of Illinois at Chicago (2018–1550) and conformed to the guidelines set forth by the Declaration of Helsinki.

### 2.2 Study design

This was a comparative, experimental study consisting of three study visits. Visit 1 determined the effect of oral vitamin C on resting vascular function, Visit 2 repeated baseline vascular function and acute inflammation was induced via vaccine, and Visit 3 determined the effect of acute inflammation and oral vitamin C during acute inflammation on vascular function. For all visits, participants arrived at the lab following an overnight fast and having abstained from exercise, caffeine, and alcohol for 24 h before each visit. All three study visits occurred at the same time of day to control for diurnal variation. Visit 2 was at least 72 h after Visit 1, and Visit 3 was exactly 24 h after Visit 2.

#### 2.2.1 Visit 1 (Rest)

After providing informed consent, participants completed a health history questionnaire. Height (cm) and weight (kg) were measured and body mass index (BMI, kg/m^2^) calculated. Body fat percentage was assessed via dual‐energy x‐ray absorptiometry (Lunar iDXA, GE). A venous blood sample was collected from the antecubital vein following standard venipuncture techniques. After a 10‐min supine rest in a temperature‐controlled room, brachial blood pressure, heart rate, and vascular function (endothelial function and arterial stiffness) were assessed. To determine the effect of vitamin C on vascular function at rest, participants then consumed 2 g of oral vitamin C (Nature Made Nutritional Products). This dose produces a physiologic level of vitamin C in plasma (Levine, Frie, et al., 1996), has been used in previous studies to improve endothelial function (Levine, Frie, et al., 1996; Raitakari et al., 2000), and reaches optimal absorption between 2 and 5 h (Levine, Conry‐Cantilena, et al., 1996; Padayatty et al., 2004). At 90‐min post ingestion, a second venous blood sample was collected. At 120‐min post ingestion, all measures were re‐assessed. After all measurements were obtained, participants completed a maximal exercise test to determine peak oxygen consumption (VO_2_peak, TrueOne, Parvo Medics, Sandy, UT) during a modified Balke treadmill protocol (3‐min warm up, self‐selected speed, +2.5% grade/2 min until 12.5%, then +0.5 mph/min to volitional exhaustion). The test was terminated and considered a maximal effort when three of the following five criteria were met: (1) respiratory exchange ratio ≥1.10; (2) final rating of perceived exertion ≥17 on the Borg scale; (3) peak heart rate within 10 beats/min of age‐predicted heart rate; (4) plateau (increase of ≤150 ml) in oxygen uptake with an increase in workload; and (5) volitional exhaustion.

#### 2.2.2 Visit 2 (Baseline)

The separate baseline from Visit 1 was obtained to avoid any acute confounding effects between vitamin C, the maximal exercise test, and the administration of the vaccine. Similar to visit 1, a venous blood sample was collected and participants rested for 10 min in the supine position in a temperature‐controlled room. Baseline brachial blood pressure, heart rate, and vascular function were then assessed prior to vaccine administration. Following, a registered nurse administered the *Salmonella typhi* polysaccharide vaccination (Typhim vi, Sanofi Pasteur SA) in the participant's nondominant arm (2 right arm, 23 left arm). This vaccine has shown to reliably increase systemic inflammation (Schroeder, Hilgenkamp, et al., 2019; Vlachopoulos et al., 2005).

#### 2.2.3 Visit 3 (Inflammation)

Participants returned to the lab 24 h later and followed the same protocol for the venous blood sample, brachial blood pressure, heart rate and vascular function assessment, and oral administration of 2 g of vitamin C as in Visit 1.

### 2.3 Measures

#### 2.3.1 Peripheral blood pressure and heart rate

Brachial systolic, diastolic, mean arterial pressure (SBP, DBP, MAP), and heart rate were assessed on the right arm in the supine position following 10 min of rest using an automated ambulatory blood pressure monitor (Mobil‐O‐Graph 24 PWA, IEM, Stolberg, Germany). Measurements were made in duplicate and averaged. If SBP or DBP differed by >5 mmHg, a subsequent measure was obtained until two values were within 5 mmHg, and those two measures were averaged.

#### 2.3.2 Brachial artery endothelial function

Brachial artery flow‐mediated dilation (FMD) was assessed as a marker of conduit artery endothelial function following standard guidelines (Thijssen et al., 2011). The right brachial artery was imaged 5–10 cm proximal to a rapid‐release blood pressure cuff via ultrasound (Hitachi‐Aloka α7, Tokyo, Japan) using a high‐frequency linear array probe. Brachial artery diameter and Doppler velocity were simultaneously measured and recorded offline for the 1‐min baseline, 5‐min vascular occlusion at 250 mmHg, and 3‐min recovery following cuff release. Brachial artery diameters were analyzed with automatic edge detection software (FMD Studio, Cardiovascular Suite, QUIPU, Pisa, Italy; Vascular Tools, Medical Imaging Applications, Coralville, IA in instances when the intima could not be removed from the image). Baseline diameter was calculated by averaging the diameter over the entire 1‐min baseline and peak diameter was selected as the highest value from 1s averages following cuff release. Absolute diameter change was determined by subtracting the baseline diameter from peak diameter during cuff release. FMD was calculated as the absolute diameter change relative to baseline and presented as a percent. Three measures were excluded due to a low‐quality FMD recording [all older adults: visit 1 baseline (n = 1), visit 1 vitamin C (n = 1), and visit 3 vitamin C (n = 1)]; and 3 individuals did not complete the vitamin C protocol during visit 1 (young adult, n = 2; older adult, n = 1). The coefficients of variation (intra‐day intraclass correlation coefficients) for baseline diameter, peak diameter, and percent FMD between Visit 1 (rest) and Visit 2 baseline for the individuals in this study were 2.2% (0.99), 2.1% (0.99), and 16.0% (0.97), respectively. Our lab coefficient of variation for FMD is 9.7%.

#### 2.3.3 Arterial stiffness

Carotid‐femoral pulse wave velocity (PWV) was assessed as a marker of aortic stiffness (Townsend et al., 2015). Arterial tonometry (NIHem, Cardiovascular Engineering, Inc.) with simultaneous ECG was used to capture carotid and femoral artery pressure waveforms on the right side of the body over a 10s epoch. PWV was calculated as the transit distance between the carotid and femoral pulse sites divided by the time delay between peak R‐wave to the foot of the corresponding pressure waveform. Transit distance was assessed as straight lines via tape measure as the distance from each tonometry recording site (femoral [d_sf_], carotid [d_sc_]) to the sternal notch and the subtraction method used (d_sf_ – d_sc_) to account for the bidirectional nature of the pressure propagation. The coefficient of variation (intra‐day intraclass correlation coefficient) for PWV between Visit 1 (rest) and Visit 2 baseline for the individuals in this study was 5.2% (0.96).

#### 2.3.4 Blood biomarkers

Blood was sampled from the antecubital vein at the beginning of each study visit after a 10–12 h fast and 90 min after oral consumption of vitamin C. Serum was obtained via a serum separation tube that was allowed to clot for 30 min. Samples were then centrifuged at 4°C for 15 min at 2000*g*. Plasma was obtained by centrifuging an EDTA tube for 10 min at 4°C and 3000*g*. Plasma and serum samples were stored at −80°C for subsequent analyses. All samples were assessed in duplicate and the coefficient of variations (CV) for duplicates for each assessment are provided below.

Interleukin‐6 (IL‐6; CV 4.3%) and C‐reactive protein (CRP; CV 4.4%) were assessed as markers of systemic inflammation in serum using commercially available high‐sensitivity enzyme‐linked immunosorbent assay (ELISA) kits (R & D Systems; Crystal Chem, Elk Grove). Vitamin C (CV, 8.0%) was assayed in plasma to determine whether acute oral vitamin C administration increased vitamin C bioavailability (CosmoBio). Serum samples were analyzed to obtain a lipid profile (low‐density lipoprotein cholesterol, high‐density lipoprotein cholesterol, total cholesterol, triglycerides) and glucose using Cholestech LDX (Cholestech Instruments).

### 2.4 Statistical analyses

All data are reported as mean and standard deviation. Normality was assessed with the Kolmogorov–Smirnov test. Data were log transformed when necessary (IL‐6, CRP) and reported as median (25^th^ percentile, 75^th^ percentile). A linear‐mixed effects model containing time (repeated), group (young vs older adults), group‐by‐time interaction, and random intercept was used to assess the change in all outcome variables separately at rest (baseline, vitamin C) and during acute inflammation (baseline, inflammation, vitamin C). The covariance structure was selected by determining the best fit according to the lowest values for the log likelihood score and information criteria after evaluation of different structures. A Bonferroni correction was applied to correct for multiple comparisons. Further, given the significant association between baseline diameter and FMD (present data, r = −0.57, *p* < 0.001), additional FMD analyses were performed including resting brachial diameter as a covariate. Statistical analyses were performed using SAS software (SAS Institute). All p‐values are two‐sided, with an a‐priori α‐level of 0.05 deemed significant.

An a priori power analysis was conducted to determine the sample size required to detect an interaction between two groups (young, older) with the vitamin C administration during acute inflammation. A medium effect (Cohen's F = 0.25) with an α of 0.05 and power of 0.80 for two groups over three measures required a total sample of 28 participants (n = 14 per group) (G*Power 3.1.9.6, Germany).

## 3 RESULTS

Recruitment goals were not achieved owing to the COVID‐19 pandemic, resulting in 9 younger and 16 older adults for the present study. Descriptive characteristics are provided in Table 1. Young and older adults had similar weight, BMI, and lipid profiles. Overall, older adults had a larger baseline brachial diameter (*p* = 0.001), lower FMD (*p* = 0.01), higher blood pressure (*p* < 0.01), and higher PWV (*p* < 0.001, Table 2). Adjusting FMD for resting brachial diameter, however, eliminated group differences (*p* = 0.28).

**TABLE 1:** Descriptive characteristics

	Young	Older	p‐value
n	9	16	
Age, yrs	24 ± 4	64 ± 5	<0.01
Male/Female	3/6	13/3	0.02
Anthropometrics			
Height, cm	168.1 ± 9.9	178.1 ± 5.2	<0.01
Weight, kg	74.4 ± 16.6	82.0 ± 11.7	0.20
BMI, kg/m^2^	26.2 ± 4.9	25.8 ± 3.2	0.81
Body fat, %	35.2 ± 6.8	31.0 ± 6.3	0.14
VO_2_peak, ml/kg/min	34.3 ± 5.6	30.9 ± 6.7	0.20
Medications			
Oral contraceptives, n (%)	3 (33)	‐‐	0.01
Hypothyroid, n (%)	2 (22)	1 (6)	0.24
Hypertension, n (%)	‐‐	1 (6)	0.44
Statin, n (%)	‐‐	2 (13)	0.27
Other, n (%)	2 (22)	3 (18)	0.84
Lipid profile			
Total Cholesterol, mg/dL	193 ± 26	205 ± 31	0.32
HDL, mg/dL	62 ± 13	67 ± 13	0.34
LDL, mg/dL	113 ± 28	120 ± 33	0.57
Triglycerides, mg/dL	92 ± 26	96 ± 37	0.76
Glucose, mg/dL	84 ± 9	83 ± 13	0.82

Note

All data presented as mean ± standard deviation or n (%).

Abbreviation: BMI, body mass index; HDL, high‐density lipoprotein; LDL, low‐density lipoprotein.

**TABLE 2:** Effect of Vitamin C on endothelial function, hemodynamics, and arterial stiffness during Visit 1 (rest)

		Baseline	Vitamin C	*p*‐value		
				Time	Group	Interaction
Baseline diameter, mm	Young	3.39 ± 0.56	3.21 ± 0.46	0.001	0.001	0.15
	Older	4.50 ± 0.81	4.39 ± 0.76			
Peak diameter, mm	Young	3.57 ± 0.54	3.44 ± 0.43	0.01	0.001	0.11
	Older	4.69 ± 0.81	4.53 ± 0.74			
Absolute diameter Δ, mm	Young	0.18 ± 0.10	0.23 ± 0.15	0.06	0.051	0.71
	Older	0.10 ± 0.09	0.14 ± 0.11			
FMD, %	Young	5.5 ± 3.4	5.7 ± 3.0	0.18	0.01	0.61
	Older	2.4 ± 1.8	3.4 ± 2.7	0.32^1^	0.28^1^	0.69^1^
SBP, mmHg	Young	116 ± 7	118 ± 6	0.10	0.01	0.58
	Older	130 ± 17	136 ± 17			
DBP, mmHg	Young	75 ± 4	75 ± 7	0.45	0.001	0.37
	Older	84 ± 7	86 ± 9			
MAP, mmHg	Young	94 ± 4	95 ± 6	0.13	0.001	0.43
	Older	105 ± 11	109 ± 11			
HR, bpm	Young	67 ± 10	67 ± 10	0.01	0.04	0.88
	Older	59 ± 10	56 ± 9			
PWV, m/s	Young	5.8 ± 0.8	5.9 ± 0.9	0.36	<0.001	0.45
	Older	9.3 ± 2.0	9.9 ± 3.0			

Note

Data presented as mean ± standard deviation.

Abbreviations: DBP, diastolic blood pressure; FMD, flow‐mediated dilation; HR, heart rate; MAP, mean arterial pressure; PWV, pulse wave velocity; SBP, systolic blood pressure.

^1^ Analysis includes covariate for resting baseline diameter.

### 3.1 Vitamin C on resting hemodynamics and vascular function (Visit 1)

**FIGURE 1:**
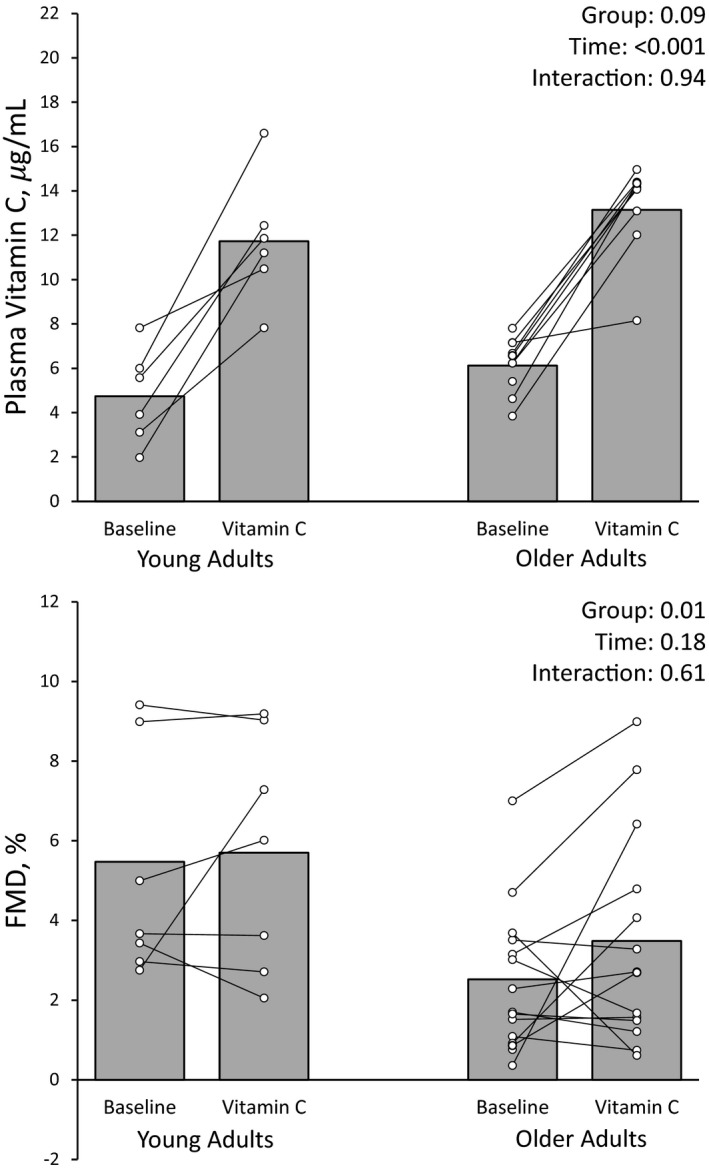
Plasma vitamin C levels and FMD following oral consumption of 2 g of Vitamin C at rest in young and older adults. FMD data are missing for 1 older adult at baseline due to a low‐quality FMD and 2 young and 1 older adult during Vitamin C due to not completing the vitamin C protocol. Plasma vitamin C data are only included for those who had data for the FMD and successful venipuncture (young adults, n = 6; older adults, n = 10)

The effect of vitamin C on resting hemodynamics and vascular function was similar in young and older adults (p for interactions >0.05). Oral consumption of vitamin C more than doubled plasma levels at 90 min similarly in younger and older adults (*p* < 0.001, Figure 1; Young 4.7 ± 2.1 to 11.7 ± 2.9 µg/ml, Older 6.2 ± 1.2 to 13.3 ± 2.1 µg/ml), however, no changes in FMD (*p* = 0.32) or PWV (*p* = 0.36) were observed. Vitamin C administration decreased baseline (*p* = 0.001) and peak (*p* = 0.01) brachial diameter, and lowered heart rate (*p* = 0.01) during Visit 1.

### 3.2 Vitamin C on hemodynamics and vascular function during acute inflammation (Visits 2 and 3)

**FIGURE 2:**
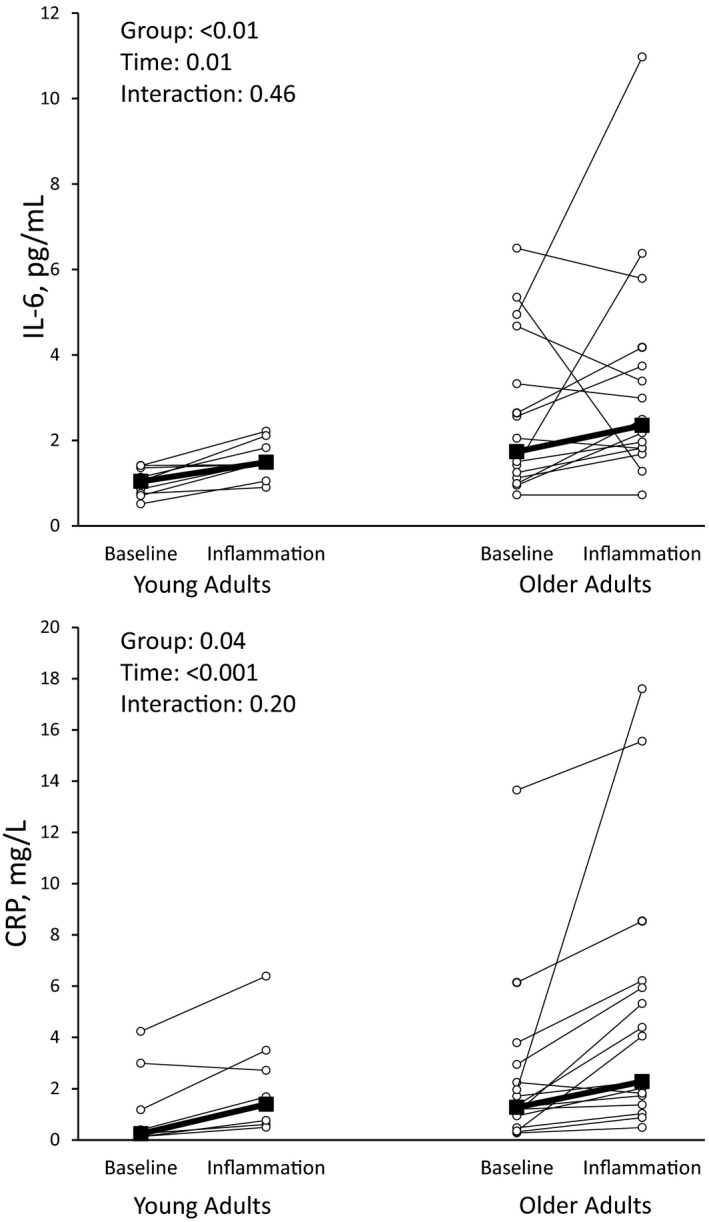
Interleukin‐6 (IL‐6) and C‐reactive protein (CRP) at baseline (Visit 2) and during acute inflammation (Visit 3)

**FIGURE 3:**
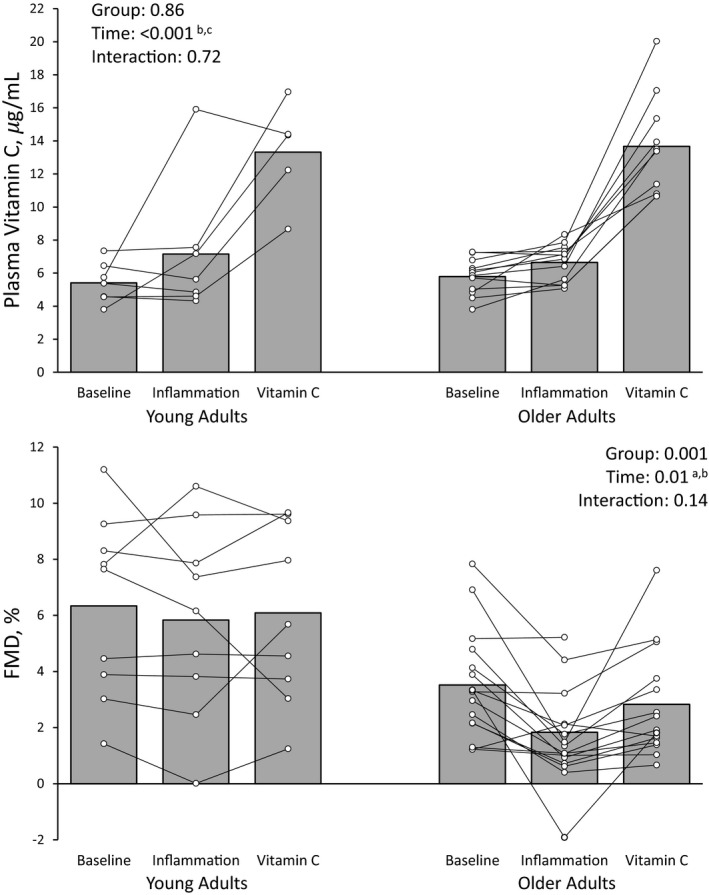
Plasma vitamin C levels and FMD during acute inflammation and following oral consumption of 2 g of Vitamin C in young and older adults. FMD data are missing for 1 older adult during vitamin C due to a low‐quality FMD. Plasma vitamin C data are included for those who had data for the FMD and successful venipuncture (young adults, n = 7; older adults, n = 12), with data missing during the vitamin C time point for 2 younger adults and 2 older adults. ^a^Baseline significantly different from inflammation, *p* < 0.05; ^b^Inflammation significantly different from vitamin C, *p* < 0.05; ^c^Baseline significantly different from vitamin C, *p* < 0.05

The vaccination induced acute inflammation, as indicated by increases in IL‐6 [*p* = 0.01, Young, 1.04 (0.75, 1.36) to 1.49 (1.43, 1.83) pg/ml; Older, 1.89 (1.18, 4.00) to 2.42 (1.82, 3.96) pg/ml] and CRP [*p* < 0.001, Young, 0.24 (0.18, 1.17) to 1.39 (0.76, 2.71) mg/L; Older 1.32 (0.71, 2.60) to 3.17 (1.55, 6.09) mg/L] in both groups at 24 h (Figure 2). Older adults had a smaller %FMD than younger adults (*p* = 0.001) and acute inflammation reduced endothelial function from Visit 2 to Visit 3 as assessed by absolute brachial artery diameter change (*p* = 0.004), and %FMD (*p* = 0.004, Table 3). Oral consumption of vitamin C during Visit 3 at least doubled plasma levels at 90 min during acute inflammation (*p* < 0.001, Figure 3) and endothelial function was restored to Visit 2 (baseline) levels. After controlling %FMD for baseline diameter, group differences were abrogated (*p* = 0.17) but the effects of acute inflammation and vitamin C remained (*p* = 0.04). Consumption of vitamin C did not significantly change IL‐6 [*p* =0.12, Young, 1.12 (1.04, 1.43) pg/ml; Older 1.93 (1.44, 2.80) pg/ml] or CRP [*p* =0.89, Young, 1.73 (1.68, 2.48) mg/L; Older, 2.26 (1.33, 4.53) mg/L]. The vascular response to acute inflammation and vitamin C was similar between young and older adults (p for interactions >0.05) and neither blood pressure (MAP, *p* = 0.23) nor PWV (*p* = 0.92) were altered in either group (*p* > 0.05).

**TABLE 3:** Effect of Vitamin C on endothelial function, hemodynamics, and arterial stiffness during acute inflammation (Visit 2 and Visit 3)

		Baseline	Inflammation	Vitamin C	*p*‐value		
					Time	Group	Interaction
Baseline diameter, mm	Young	3.36 ± 0.51	3.38 ± 0.58	3.35 ± 0.61	0.08	<0.001	0.28
	Older	4.54 ± 0.77	4.53 ± 0.77	4.45 ± 0.78			
Peak diameter, mm	Young	3.57 ± 0.50	3.57 ± 0.54	3.54 ± 0.55	0.03^c^	<0.001	0.19
	Older	4.69 ± 0.74	4.60 ± 0.78	4.57 ± 0.77			
Absolute diameter Δ, mm	Young	0.20 ± 0.10	0.19 ± 0.10	0.20 ± 0.87	0.004^a^, ^b^	0.01	0.05
	Older	0.15 ± 0.07	0.07 ± 0.06	0.12 ± 0.07			
FMD, %	Young	6.3 ± 3.3	5.8 ± 3.4	6.1 ± 3.2	0.01^a^, ^b^	0.001	0.14
	Older	3.5 ± 1.9	1.6 ± 1.7	2.8 ± 1.9	0.04^1^	0.17^1^	0.12^1^
SBP, mmHg	Young	114 ± 8	115 ± 8	117 ± 8	0.28	0.01	0.97
	Older	127 ± 13	127 ± 13	129 ± 15			
DBP, mmHg	Young	73 ± 4	72 ± 3	73 ± 4	0.46	<0.001	0.75
	Older	83 ± 8	83 ± 9	85 ± 8			
MAP, mmHg	Young	92 ± 5	92 ± 5	93 ± 4	0.23	0.001	0.95
	Older	103 ± 10	103 ± 9	105 ± 9			
HR, bpm	Young	67 ± 10	70 ± 6	65 ± 8	0.001^b^, ^c^	0.19	0.46
	Older	62 ± 14	63 ± 15	58 ± 13			
PWV, m/s	Young	5.6 ± 0.4	5.6 ± 0.6	5.7 ± 0.5	0.92	<0.001	0.99
	Older	8.8 ± 2.1	8.8 ± 1.7	8.9 ± 2.1			

Note

Data presented as mean ± standard deviation.

Abbreviations: DBP, diastolic blood pressure; FMD, flow‐mediated dilation; HR, heart rate; MAP, mean arterial pressure; PWV, pulse wave velocity; SBP, systolic blood pressure.

^1^ Analysis includes covariate for resting baseline diameter.

^a^ Baseline significantly different from inflammation, *p* < 0.05.

^b^ Inflammation significantly different from vitamin C, *p* < 0.05.

^c^ Baseline significantly different from vitamin C, *p* < 0.05.

## 4 DISCUSSION

The present study confirms our previous findings that the administration of a vaccine induces systemic inflammation and reduces endothelial function (Lane‐Cordova, Ranadive, et al., 2016; Schroeder, Hilgenkamp, et al., 2019). The endothelial response to acute inflammation as assessed by brachial FMD does not appear to be influenced by age, aligning with some (Lane‐Cordova, Ranadive, et al., 2016) but not all (Lane‐Cordova, Phillips, et al., 2016) previous findings. Furthermore, acute inflammation induced by vaccine‐mediated immune response did not change arterial stiffness assessed by PWV in either young or older adults. Acute administration of the oral antioxidant vitamin C, however, restored brachial FMD in young and older adults. These data suggest that reduced endothelial function during acute inflammation (induced systemically) in both young and older adults may be related to changes in antioxidant capacity and/or potential alterations in nitric oxide bioavailability.

### 4.1 Vitamin C on resting vascular function

We did not observe an effect of vitamin C at baseline (no acute inflammation) on endothelial function or arterial stiffness. Data indicate acute administration of oral vitamin C improves endothelial function in populations with, or at risk for, disease (coronary artery disease (Levine, Frie, et al., 1996), young smokers (Raitakari et al., 2000)). Oral vitamin C is less effective, however, in middle‐age or older adults who are healthy (Eskurza et al., 2004a) or have large (>75^th^ percentile (Holder et al., 2021)) baseline FMDs (Duffy et al., 2001). The null effect of vitamin C on FMD may therefore not be surprising considering the relatively good health of the individuals enrolled in the present study who are not at high risk for cardiovascular disease, despite their lower baseline FMDs compared to recently published reference ranges in a smaller number of older adults (Holder et al., 2021). Similarly, vitamin C appears to lower arterial stiffness assessed as pulse wave velocity in high‐risk populations, such as heart failure (Nightingale et al., 2003), but has minimal effect on arterial stiffness in healthy adults (Eskurza et al., 2004b; Kelly et al., 2008; Nightingale et al., 2003). Supraphysiologic infusions of vitamin C did not improve aortic stiffness in healthy young and older men (Eskurza et al., 2004a), similar to our findings with oral vitamin C. Modulation of local arterial stiffness (e.g., carotid compliance) via vitamin C, however, may differ by sex and age (Eskurza et al., 2004a; Moreau et al., 2005). Ultimately, we likely saw no benefit of vitamin C on vascular function under resting conditions because administration of an oral antioxidant appears only effective in high‐risk populations.

### 4.2 Vitamin C on vascular function during acute inflammation

Reductions in endothelial function were observed during acute inflammation in both young and older adults in the present study (time effect, *p* = 0.01). These results replicate prior studies in both young (Hingorani et al., 2000a; Schroeder, Hilgenkamp, et al., 2019; Wallace et al., 2010) and older (Antoniades et al., 2011; Lane‐Cordova, Ranadive, et al., 2016) adults that also observed impairment in FMD during acute inflammation induced via vaccination, however, the response in older adults is not reported as consistently in the literature (Lane‐Cordova, Phillips, et al., 2016; Ranadive et al., 2014). Despite using a similar time course (24 h post), the discrepancy in findings within the older adult population may be related to the inflammatory stimulus used (*Salmonella typhi* vs. influenza vaccine) or the differences in health and fitness of the older adults within each study.

To our knowledge, our data are the first to suggest the oral consumption of 2 g of vitamin C can restore endothelial function during acute inflammation in both young and older adults. We did not observe a differential FMD response between the young and older adults (p for interaction = 0.14), however, it should be noted that our young group was underpowered. Thus, there could be differential responses across groups. The improvement in FMD with vitamin C during acute inflammation may suggest that vitamin C altered the vascular antioxidant balance or improved nitric oxide bioavailability (May & Harrison, 2013). Our findings in older adults align with data in young adults that restored FMD using supraphysiologic infusions of vitamin C, of which further suggested reductions in FMD stem from elevated oxidative stress and reduced nitric oxide bioavailability (Clapp et al., 2004; Mittermayer et al., 2005; Pleiner et al., 2002). Interestingly, we observed an improvement in FMD with only an approximately twofold increase in systemic vitamin C whereas the prior studies utilizing the supraphysiologic infusions elicited an approximately sixfold increase in systemic vitamin C (Mittermayer et al., 2005; Pleiner et al., 2002). Although we did not assess nitric oxide bioavailability, vitamin C at lower doses may stabilize tetrahydrobiopterin (BH4) levels, an essential co‐factor for the production of nitric oxide (Huang et al., 2000; Trinity et al., 2016). This hypothesis is in line with a previous study conducted by Mittermayer et al. (2005) who found that administration of both BH4 or vitamin C in young healthy men maintained the forearm blood flow response to acetylcholine during endotoxin, another methodological approach to induce acute inflammation. Together, these data support the idea that reductions in endothelial function during acute inflammation could stem from altered nitric oxide bioavailability and acute administration of vitamin C can restore endothelial function during acute inflammation in both young and older adults.

Interestingly, we observed improvements in endothelial function with vitamin C despite observing no changes in markers of inflammation. These results may suggest that although the inflammatory response to the vaccine is the instigator of the changes in endothelial function, the expected subsequent vascular oxidative stress is likely the primary cause of the reduction in endothelial function. A previous study using salsalate (a nuclear transcription factor nuclear factor κB (NFκB)‐inhibitor), also supports this hypothesis (Pierce et al., 2009). Pierce et al. (2009) observed improvements in FMD following salsalate administration for 4 days without changes in inflammatory markers, and additionally, the infusion of vitamin C improved FMD in the placebo group with no further improvements in FMD observed in the salsalate group. This data suggests manipulating oxidative stress can improve endothelial function without altering inflammatory markers.

We did not observe any significant changes in arterial stiffness in the present study during acute inflammation. Acute inflammation may reduce bioavailability of the potent vasodilator nitric oxide and subsequently functionally stiffen large, elastic arteries (Jain et al., 2014; Vlachopoulos et al., 2005). Other previous studies in both young and older adults have shown increases in pulse wave velocity (Jae et al., 2013; Vlachopoulos et al., 2005; Wallace et al., 2010), however, we have not previously replicated these results within our laboratory (Lane‐Cordova, Phillips, et al., 2016; Schroeder, Lefferts, et al., 2019). The differences between the previous studies and ours may partially relate to the amount of time between the induction of acute inflammation and the changes in the inflammatory profile. Both Wallace et al. (2010) and Vlachopoulos et al. (2005) observed increases in PWV at 8h following vaccination in young, healthy adults, which occurred at a time with a large increase in IL‐6 (~3–6 pg/ml) and minimal change in CRP (~0.2 mg/L). In our study, we see modest increases in IL‐6 (~0.5 pg/ml) accompanied by much larger changes in CRP (~1.5 mg/L) at 24 h, thus, the timing and concomitant changes in the inflammatory profile may partially explain the differences between studies. It should be noted, however, that Jae et al. (2013) still observed increases in PWV at 24 h following vaccination in older adults, despite having more comparable changes in inflammatory profiles as our data.

We additionally hypothesized that alterations in nitric oxide bioavailability during acute inflammation may functionally stiffen the aorta and would subsequently be restored with vitamin C. With no changes in PWV during acute inflammation, it is not surprising that we did not observe any changes in PWV with the administration of vitamin C (i.e., there was no apparent large artery stiffening present for vitamin C to improve). Acute (functional) changes in arterial stiffness are also seen with changes in blood pressure, that is, distending pressure. We did not observe any changes in mean arterial pressure either during inflammation or in response to vitamin C, eliminating another mechanism by which we may have observed changes in PWV. Overall, vitamin C did not alter aortic stiffness in our population and may again reflect their general overall good health (Eskurza et al., 2004b; Kelly et al., 2008; Nightingale et al., 2003).

### 4.3 Study strengths and limitations

To our knowledge, we are the first study demonstrating that vitamin C reverses vascular dysfunction induced by acute inflammation in older adults. Additionally, in comparison to previous acute inflammation studies, we have administered an oral dose of vitamin C. Our oral dose is more practical in a real‐world setting than a supraphysiologic infusion, making these results easier to disseminate and translate to the general population.

Our study is not without limitations. The smaller sample size in the present study due to the COVID‐19 pandemic may be a limitation as we did not meet the required sample size in the young adult group based on our a priori power analyses. Moreover, our groups were not well matched by sex. Future studies with larger sample sizes will be necessary to confirm our results. Additionally, we allowed medications within our groups. Although some medications (i.e., for hypertension, hypercholesterolemia, etc.) may influence our outcomes of interest, the inclusion of individuals with medications increases the generalizability of our findings. Furthermore, a previous study from our laboratory has shown that the medication status did not alter the FMD response during acute inflammation (Lane‐Cordova, Ranadive, et al., 2016). We are unable to establish the mechanism by which vitamin C altered vascular function because we did not measure a complete panel of biomarkers indicative of aberrant reactive oxygen or reactive nitrogen species modification of proteins and lipids. Future investigations should incorporate more indices of nitric oxide bioavailability (e.g., nitric oxide, nitrotyrosine, BH4, etc.) and oxidative stress to truly determine the effect of vitamin C on oxidative stress and nitric oxide bioavailability. We also did not include a placebo control group without vitamin C treatment to confirm all changes in endothelial function were related to the vitamin C consumption, and noted an unanticipated reduction in brachial diameter with vitamin C administration during Visit 1. The cause of this is unknown but may reflect measurement error or inconsistency and not be physiological in nature given it occurred in both groups and was not apparent during Visit 3.

Last, we assessed the effect of acute inflammation on vascular function and two markers of inflammation (IL‐6 and CRP) 24 h following vaccine administrations. Although not the first study to use this time frame (Lane‐Cordova, Phillips, et al., 2016; Ranadive et al., 2014; Schroeder, Hilgenkamp, et al., 2019), other studies have also observed reductions in endothelial function at 8 h post‐vaccine (Hingorani et al., 2000b; Vlachopoulos et al., 2005). The time course of the inflammation is important to consider when interpreting the results. Although we have observed increases in both IL‐6 and CRP at 24 h, IL‐6 is a precursor for the initiation and production of CRP. In the study by Vlachopoulos et al. (2005), the greatest increase in IL‐6 was at 8 h post‐vaccination, whereas CRP was greatest at the 32 h time point. Thus, the timing of our vascular assessments likely reflects increasing CRP, as the peak of our IL‐6 response probably occurred before the 24‐hour time point. Despite the variation in the markers of inflammation, it is evident our inflammatory stimulus altered vascular function and likely nitric oxide bioavailability.

### 4.4 Implications

During the acute inflammation, we observed a reduction in FMD, on average, of 0.5% in the young adults and 1.9% in the older adults. This large reduction in FMD, especially in older adults, is clinically relevant as previous meta‐analyses suggest a 1% decrease in FMD is associated with an 8% increase in the risk of a cardiovascular event (Inaba et al., 2010). Interestingly, the vitamin C increased FMD by 0.3% and 1.2% on average in the young and older adults, respectively. The administration of vitamin C therefore led to FMD only being 0.2% and 0.7% lower in the young and older adults, respectively. Although unable to be determined in the present study, in the older adults in particular, the much smaller change from baseline observed during vitamin C (−0.7%) compared to when no intervention is administered (−1.9%) may impact cardiovascular risk during acute inflammatory insults.

## 5 CONCLUSION

In conclusion, our data suggest that oral vitamin C exerts beneficial effects and restores endothelial function during acute inflammation in young and older adults. Vitamin C may therefore be a practical future therapy for reducing cardiovascular event risk in older adults during acute inflammation, perhaps due to its vascular antioxidant capacity or its possible additional ability to alter nitric oxide bioavailability by stabilizing BH4. Further research in this area is necessary to confirm its utility, especially in older adults with greater cardiovascular risk, and mechanism.

## CONFLICT OF INTEREST

No conflict of interest, financial or otherwise, are declared by the author(s).

## AUTHOR CONTRIBUTION

Elizabeth C. Lefferts and Bo Fernhall conception and design of research; Elizabeth C. Lefferts, Brooks A. Hibner, Wesley K. Lefferts, and Natalia S. Lima performed experiments; Elizabeth C. Lefferts analyzed data; Elizabeth C. Lefferts, Brooks A. Hibner, Wesley K. Lefferts, Natalia S. Lima, Tracy Baynard, Jacob M. Haus, Abbi D. Lane‐Cordova, Shane A. Phillips, and Bo Fernhall interpreted results; Elizabeth C. Lefferts drafted manuscript and prepared figures; Elizabeth C. Lefferts, Brooks A. Hibner, Wesley K. Lefferts, Natalia S. Lima, Tracy Baynard, Jacob M. Haus, Abbi D. Lane‐Cordova, Shane A. Phillips, Bo Fernhall. edited and revised manuscript; Elizabeth C. Lefferts, Brooks A. Hibner, Wesley K. Lefferts, Natalia S. Lima, Tracy Baynard, Jacob M. Haus, Abbi D. Lane‐Cordova, Shane A. Phillips, Bo Fernhall approved final version of manuscript.
